# Cancer-derived exosomal miR-7641 promotes breast cancer progression and metastasis

**DOI:** 10.1186/s12964-020-00700-z

**Published:** 2021-02-22

**Authors:** Songjie Shen, Yu Song, Bin Zhao, Yali Xu, Xinyu Ren, Yidong Zhou, Qiang Sun

**Affiliations:** 1 Peking Union Medical College Hospital, Chinese Academy of Medical Sciences and Peking Union Medical College, No. 1 Shuaifuyuan, Dongcheng District, Beijing, 100730 China; 2 Department of Pathology, Peking Union Medical College Hospital, Chinese Academy of Medical Sciences and Peking Union Medical College, Beijing, China

**Keywords:** Breast cancer, Exosome, microRNA, Tumor progression, Metastasis

## Abstract

**Background:**

Intercellular communication is crucial for breast cancer progression and metastasis. However, the role of cancer-derived exosomes and their crucial microRNA (miRNA) cargoes mediating intercellular communication requires further investigation.

**Methods:**

Cancer-derived exosomes were isolated using differential centrifugation and differentially expressed miRNAs were determined by microarrays and qRT-PCR analysis. Cell proliferation, wound-healing, Transwell invasion, and tumor xenograft assays were used for functional research. Plasma exosomal RNA was isolated to verify its role as a prognostic biomarker.

**Results:**

We found that the tumor-promoting capacity of the exosomes was positively related to their cells of origin. MiR-7641 was identified to be the most differentially expressed miRNA, both at endogenous and secretory levels in high-metastatic cancer cells. MiR-7641 could promote tumor cell progression and metastasis, and that these functions of miR-7641 could alter recipient cells via transportation of exosomes. Additionally, exosomal miR-7641 could promote tumor growth in vivo; and its levels were significantly elevated in the plasma of patients with distant metastasis. Bioinformatics analysis has suggested that miR-7641 is correlated with breast cancer survival, and several important cellular and biological processes are closely targeted by miR-7641.

**Conclusion:**

The findings indicate miR-7641 to be an important component of the cancer exosomes in promoting tumor progression and metastasis via intercellular communication. Additionally, exosomal miR-7641 may serve as a promising non-invasive diagnostic biomarker and potential targetable candidate in breast cancer treatment.

**Video Abstract**

## Background

Breast cancer is the most commonly diagnosed cancer in women worldwide[1, 2]. Despite improvements in local and systemic therapies, metastatic breast cancer remains incurable and is the major cause of cancer-related deaths[3]. Cancer progression and metastasis includes multiple steps. Moreover, evidence suggests that intercellular communication is crucial during these steps[4, 5]; although, the underlying mechanisms remain poorly understood.

Exosomes are extracellular vesicles with an endosomal origin and a size range of 40–160 nm in diameter[5]. The physiological purpose for cancer cells to secrete exosomes remains largely unknown and needs investigation; however, recent studies indicate a functional and selective accumulation of cellular bioactive components in the exosomes, suggesting their involvement in cell-to-cell communication during tumor progression and metastasis[5–7]. Several studies showed that exosomes released from highly metastatic cancer cells could be taken up by other cells, and thus transfer metastatic capability to non-metastatic cells[8, 9].

MicroRNAs (miRNAs) are small, non-coding RNAs that can post-transcriptionally repress the activity of specific target mRNAs. More than half of all the mRNAs are estimated to be targets of miRNAs, and each miRNA is predicted to regulate about hundreds of target mRNAs[10, 11]. There is considerable evidence indicating that miRNAs are involved in the progression and metastasis of cancer cells via epigenetic regulations[12]. Further, miRNAs can be encapsulated in exosomes. Several studies have reported that certain miRNAs are enriched in the exosomes compared to their cells of origin, and that exosomes derived from metastatic breast cancer cells showed enrichment of miRNAs compared with exosomes derived from non-metastatic breast cancer cells[13, 14]. Thus, exosomes may function as a vehicle for intercellular miRNA transfer (“a message in a bottle”) and mode of intercellular communication[15]. However, which are the key miRNAs transferred as cargoes via cancer-derived exosomes in promoting tumor proliferation and metastasis remains unclear.

In the present study, we investigated the characteristics and functional heterogeneity between exosomes derived from breast cancer cells with different metastatic abilities. We hypothesized that miRNAs transferred via exosomes could be essential in exerting epigenetic alterations in recipient cells, and identified the critical cancer-derived exosomal miRNAs. Furthermore, we also confirmed that cancer-derived exosomal miRNAs in plasma could serve as a biomarker for assessing breast cancer metastasis. Thus, our study would help elucidate the mechanisms of breast cancer metastasis mediated via exosomes, and offer additional opportunities for potential therapeutic targets and prognostic biomarkers.

## Materials and methods

### Specimens and primary cells

Human plasma specimens were obtained from 28 patients with breast cancer (15 without distant metastasis and 13 with distant metastasis) treated at the Peking Union Medical College Hospital (PUMCH). The human primary breast cancer cells were isolated from fresh tumor tissues of five patients who underwent surgery at the PUMCH. After culturing them in Dulbecco’s modified Eagle’s Medium (DMEM) (Hyclone, Logan, UT, USA) containing Rho kinase inhibitor (Y-27632) and fibroblast feeder cells for several passages[16], the human primary breast cancer cells were cultured in DMEM supplemented with 10% fetal bovine serum (FBS) (Gibco, Carlsbad, CA, USA) for further experiments.

### Cell culture and transfection

Human breast cancer cell lines—MCF-7, MDA-MB-231, and HCC-1937—were obtained from Cell Bank of the Chinese Academy of Sciences (Shanghai, China), and authenticated by short tandem repeat profiling. MCF-7 and MDA-MB-231 cells were cultured in DMEM, while HCC-1937 cells were grown in RPMI 1640 medium (Hyclone). All the cell lines were cultured in media supplemented with 10% FBS at 37 °C with 5% CO_2_ in a humidified incubator. For experiments related to exosomes, the serum added to the cell culture medium was depleted of indigenous exosomes by ultracentrifugation at 120,000×*g* for 8 h at 4 °C. The miR-7641 mimics, miR-7641 inhibitors, and corresponding control miRNAs (miRNA negative control, NC) were designed and synthesized by General Biosystems (Anhui, China). Cells were seeded into 6-well plates and grown to 50–60% confluence. Further, cells were transfected using Lipofectamine 2000 (Invitrogen, Grand Island, NY, USA) in Opti-MEM (Invitrogen) according to the manufacturer’s instructions. After incubating for 48 h, the RNA levels were assessed using qRT-PCR.

### Exosome isolation from tumor cells

For exosome isolation, cells were cultured for 48 h in respective medium supplemented with exosome-free serum that was prepared by centrifuging at 12,000 × g for 8 h. Exosomes were purified from the conditioned medium using differential centrifugation as described earlier with minor modifications[17]. Briefly, the collected culture mixture was pre-cleared by centrifugation at 500 × g for 10 min and then at 20,000 × g for 20 min. The supernatant was further filtered using a 0.22-μM filter (Millipore, MA, USA). Next, exosome pellets were harvested by centrifugation at 100,000 × g for 2 h (Optima MAX-XP, Beckman Coulter, rotor TLA 100.3). Exosome pellets were resuspended in 100 µl of cold phosphate-buffered saline (PBS). Vesicular protein concentration was measured using the Pierce BCA protein assay kit (Thermo Fisher Scientific, MA, USA). For cell treatment, 2 μg of exosomes were added to 2 × 10^5^ recipient cells and incubated for 24 h.

### Transmission electron microscopy

For transmission electron microscopy (TEM), approximately 10 µl aliquot of exosomes was placed on a copper mesh and incubated at 20 °C for 10 min. After washing with sterile distilled water, the exosomes were contrasted using uranyl-oxalate solution for 1 min. Further, the sample was dried for 2 min under incandescent light. The copper mesh was examined at 100 kV using a JEOL JEM-1400Plus TEM (JEOL, Peabody, USA) according to the manufacturer’s instructions.

### Protein extraction and western blot analysis

Total protein was extracted using a cell lysis reagent, and concentration was measured using the Pierce BCA protein assay kit (Thermo Fisher Scientific, Waltham, MA, USA). Protein samples were separated by 10% sodium dodecyl sulfate polyacrylamide gel electrophoresis. The separated proteins were transferred onto polyvinylidene difluoride membrane (Millipore, MA, USA), which were then blocked with 5% skim milk powder suspended in Tris-buffered saline-Tween. After incubation for 1 h, the membrane was incubated with primary antibodies at 4 °C for 4 h. The primary antibodies used in the study were rabbit anti-CD9 (ab92726, 1:1000, Abcam, MA, USA), rabbit anti-CD63 (A5271, 1:1000, ABclonal Technology, China), and rabbit anti-tubulin (ab6046, 1:1000, Abcam). The membranes were then incubated for 1 h with goat anti-rabbit secondary antibody (ab205718, 1:5000, Abcam). Proteins were detected with an ECL detection Kit (Millipore, MA, USA) using the Tanon-5200 Imaging System (Tanon, Shanghai, China).

### Cell wound-healing assay

For cell wound-healing assay, 5 × 10^5^ cells were seeded in 6-well plates. The cells were scratched with a sterile tip perpendicular to the previously painted line. The wounds of scratch were photographed, and cell migration was measured at indicated time points of 0 and 24 h using the BX41 light microscope.

### Transwell invasion assay

Cell invasion were assessed by the 24-well Transwell chambers (Costar, NY, USA) according to the manufacturer’s instructions. Transwell inserts were pre-coated with 100 µl of Matrigel (BD Biosciences, CA, USA). Briefly, the same amount of cells from different groups were suspended in serum-free medium and plated in each of the upper chambers; bottom chambers were filled with growth medium containing 10% FBS. After incubation for 24 h, the invaded cells at the bottom surface were fixed and stained according to the manufacturer’s instructions. Representative fields were photographed and the number of invaded cells per field were counted using the BX41 light microscope.

### RNA extraction and quantitative real-time PCR (qRT-PCR)

Total cellular and exosomal RNA was extracted using the TRIzol reagent (Invitrogen, Carlsbad, CA, USA) as per the manufacturer’s protocol. Reverse transcription was performed using the Superscript III (Invitrogen) with random primers. Real-time PCR analysis was performed on the ABI 7500 instrument (ABI Inc., USA) with 20-µl reaction volumes containing 1 µl reverse transcription product, 10 µl 2X SYBR Green Mix (Invitrogen), 0.8 µl paired specific primers (10 µM), and 8.2 µl H_2_O. The *U6* small nuclear RNA was used as an internal control, and cel-miR-39 was used as an external control. The primers used for qRT-PCR analysis were purchased from RiboBio (Guangzhou, China) and listed in Supplementary Table S1. The relative levels of each miRNA were calculated using the 2^−ΔΔCT^ method, where CT was the difference in threshold cycles to detect fluorescence.

### Microarray analysis of cellular and exosomal miRNAs

RNA was isolated from MCF-7 and MDA-MB-231 cells and from exosomes isolated from their respective conditioned medium using the TRIzol reagent (Invitrogen, Carlsbad, CA, USA) according to the manufacturer's protocol. Microarray analysis of cellular and exosomal miRNAs was performed at the Shanghai Genechem Biotechnology (Shanghai, China) using the Affymetrix Genechip miRNA 4.0 (Affymetrix, Santa Clara, CA, USA). The quantity and quality of the miRNA extractions was determined using the Agilent Bioanalyzer 2100 (Agilent Technologies, Columbia, MD) and Nanodrop 2000 (Thermo Fisher Scientific, Waltham, MA, USA). Only samples with RNA Integrity Number > 7.0, 28S/18S ribosomal (r) RNA ratio > 0.7, and absorbance (A) 260 / A280 ratio in 1.7–2.2 range were processed for microarray analysis. Total RNA was labeled with Biotin using a FlashTag Biotin HSR RNA Labeling kit (Genisphere, Hatfield, PA, U.S.) and hybridized overnight with the GeneChip Hybridization Oven 645. The GeneChip miRNA arrays were washed and stained using the Affymetrix GeneChip Hybridization Wash and Stain Kit, and then scanned with the Affymetrix GeneChip Scanner 3000. For analysis of the miRNA array, CEL-files of the raw data were produced using the Affymetrix GeneChip Command Console Software Version 4.0. The microarray expression data for miRNAs was deposited at the Gene Expression Omnibus data repository (accession number: GSE154054).

### Cell proliferation assay

In vitro cell proliferation assay was measured using the Cell Counting Kit-8 (CCK-8, Dojindo, Kumamoto, Japan). Cells were seeded into 96-well plates and cultured overnight. At 24, 48, and 72 h after respective treatments, culture media was removed, and cells were incubated with 100 µl of CCK-8 solution for 1 h. The optical density of each well was measured using a microplate spectrophotometer (Pulang Technology, China) at 450 nm (optical density, OD).

### In vivo experiments

For in vivo tumor xenograft assays, 15 female BALB/c nude mice (5-weeks-old) were purchased from Cavens (Jiangsu, China) and housed for 1-week before injection. A total of 5 × 10^6^ MDA-MB-231 cells were injected subcutaneously into the right mammary fat pads of each mouse. Further, 15 mice were randomly divided into three groups (*N* = 5, each) and intravenously injected with 6 μg of exosomes from different tumor cells twice every week. The mice were physically examined every week. After 4-weeks, mice were sacrificed and tumors were excised. All procedures involving animals were monitored in accordance with the ethical standards and the Care and Use of Laboratory Animal guidelines issued by the administrative government, under the protocol approved by the Institutional Animal Care and Use Committee of PUMCH.

### Plasma exosomal RNA isolation and RNA extraction

Peripheral blood samples from patients with breast cancer were collected in EDTA tubes following a regular venipuncture procedure. After centrifugation at 3,000 × g for 15 min at 4 °C, the plasma was aspirated, and stored at − 80 °C till use. Plasma exosomal RNA isolation was optimized according to the method previously described[18]. Briefly, plasma sample was diluted using PBS, centrifuged at 10,000 × g for 30 min, and filtered through a 0.22 μm filter (Millipore, MA, USA) to remove any cell debris. The supernatant was ultracentrifuged at 100,000 × g, at 4 °C for 2 h to pellet the exosomes. After washing with PBS, the exosome pellet was re-suspended in 100 µl PBS. Total RNA was extracted from plasma-derived exosomes using miRNeasy Serum/Plasma Kit (Qiagen, Germantown, MD, USA) following the manufacturer’s instructions.

### Target prediction and bioinformatic validation of miR-7641

The target genes of miR-7641 were predicted using TargetScan (http://www.targetscan.org/vert_71/), miRWalk (http://mirwalk.umm.uni-heidelberg.de/), and miRanda (http://www.miranda.org/) algorisms. Target genes which were predicted by all three databases were finally included. GO and KEGG enrichment analyses were conducted on the predicted target genes, and the results of the enrichment analysis were displayed with bubble plots. The miR-7641 expression data of breast cancer patients and the matched follow-up data were retrieved from the TCGA database (https://tcga-data.nci.nih.gov), and survival analysis were performed on the disease-free survival (DFS) of breast cancer patients.

### Statistical analyses

Each experiment was repeated at least three individual times, unless otherwise indicated, and all results were presented as mean ± SD. The Fisher’s exact test and the two-tailed Student’s *t*-test were performed to analyze statistical significance. The One-way ANOVA was used for assessing comparisons between more than 2 groups. Kaplan–Meier method was used for survival analysis. All statistical analyses were performed using SPSS version 25.0 (IBM Corp. Armonk, NY, USA) or Graphpad Prism version 8.0 (Graphpad Software, CA, USA). *p-*values < 0.05 were considered statistically significant.

## Results

### Isolation and characterization of exosomes released from tumor cells

**Fig. 1:**
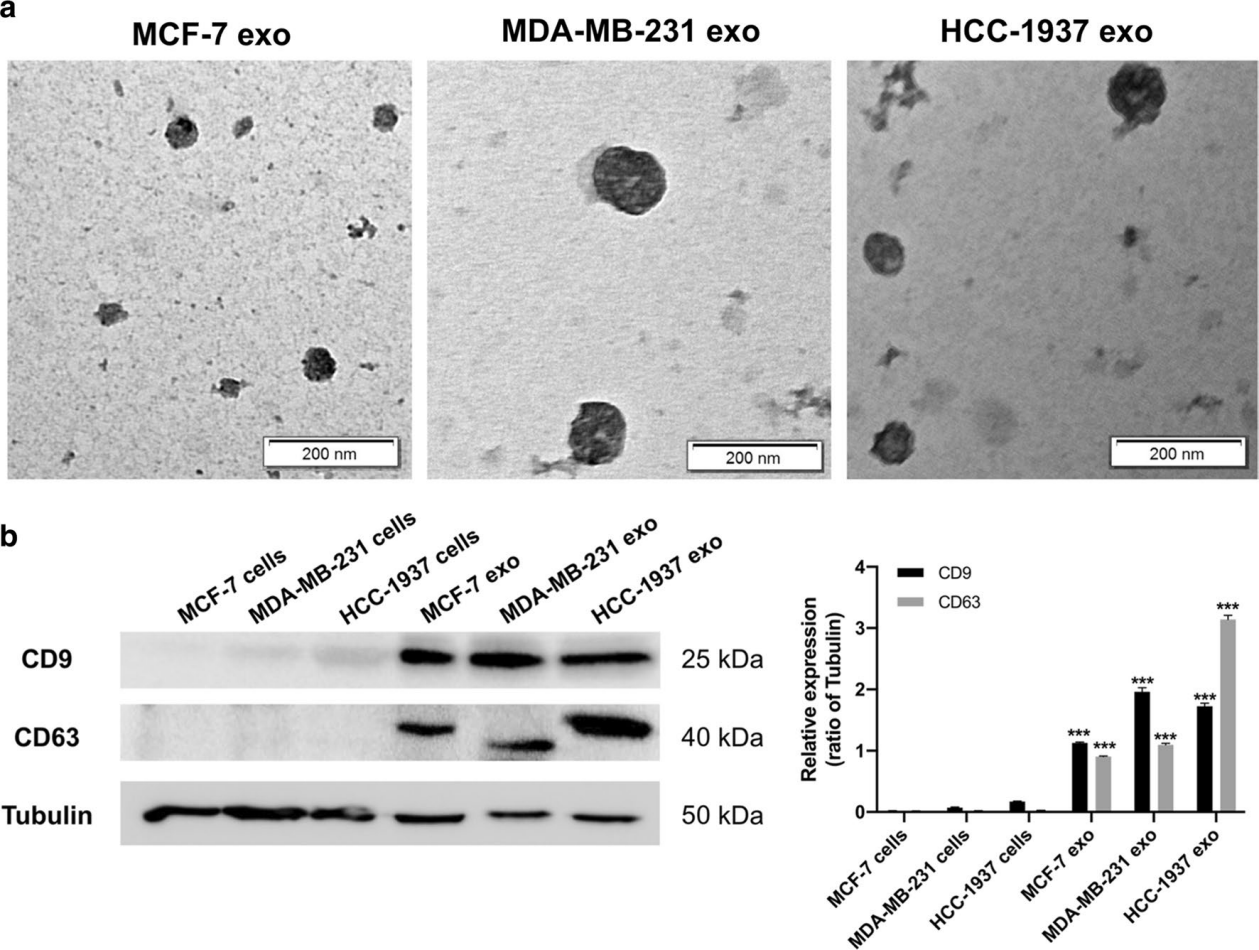
Identification of exosomes released by different breast tumor cells.** a** Transmission electron microscopy analysis of exosomes isolated from conditioned media of MCF-7, HCC-1937, and MDA-MB-231 cells. **b** Western blotting analysis of exosomal protein markers (CD9 and CD63) in tumor cells and their respective purified exosomes. Each experiment was performed at least three independent times and results were presented as mean ± SD. Scale bar: 200 nm, ***p < 0.001 compared with their respective cells of origin

Exosomes in conditioned media were isolated using differential centrifugation from three breast cancer cell lines (MCF-7, HCC-1937, and MDA-MB-231). To determine the morphology, the exosomes were resuspended in PBS and then examined by TEM. The exosomes isolated from these cells showed a uniformly cup-shaped structure with a characteristic diameter range of 50–120 nm (Fig. 1a). However, exosomes secreted from highly-metastatic cancer cell lines (HCC-1937 and MDA-MB-231) were larger in size than those from less-metastatic cancer cells (MCF-7) (Fig. 1a). To further verify that the isolated pellets were exosomes, we analyzed levels of the exosome-specific markers (CD9 and CD63) in tumor cells and pellets isolated from their respective media. As shown in Fig. 1b, levels of CD9 and CD63 were very low in cell lysates of the three cell lines, but were significantly elevated in isolated exosome pellets. These results suggest that the pellets isolated using differential centrifugation from conditioned media were exosomes secreted by tumor cells.

### Exosomes from metastatic cells could promote tumor cell migration and invasion

**Fig. 2:**
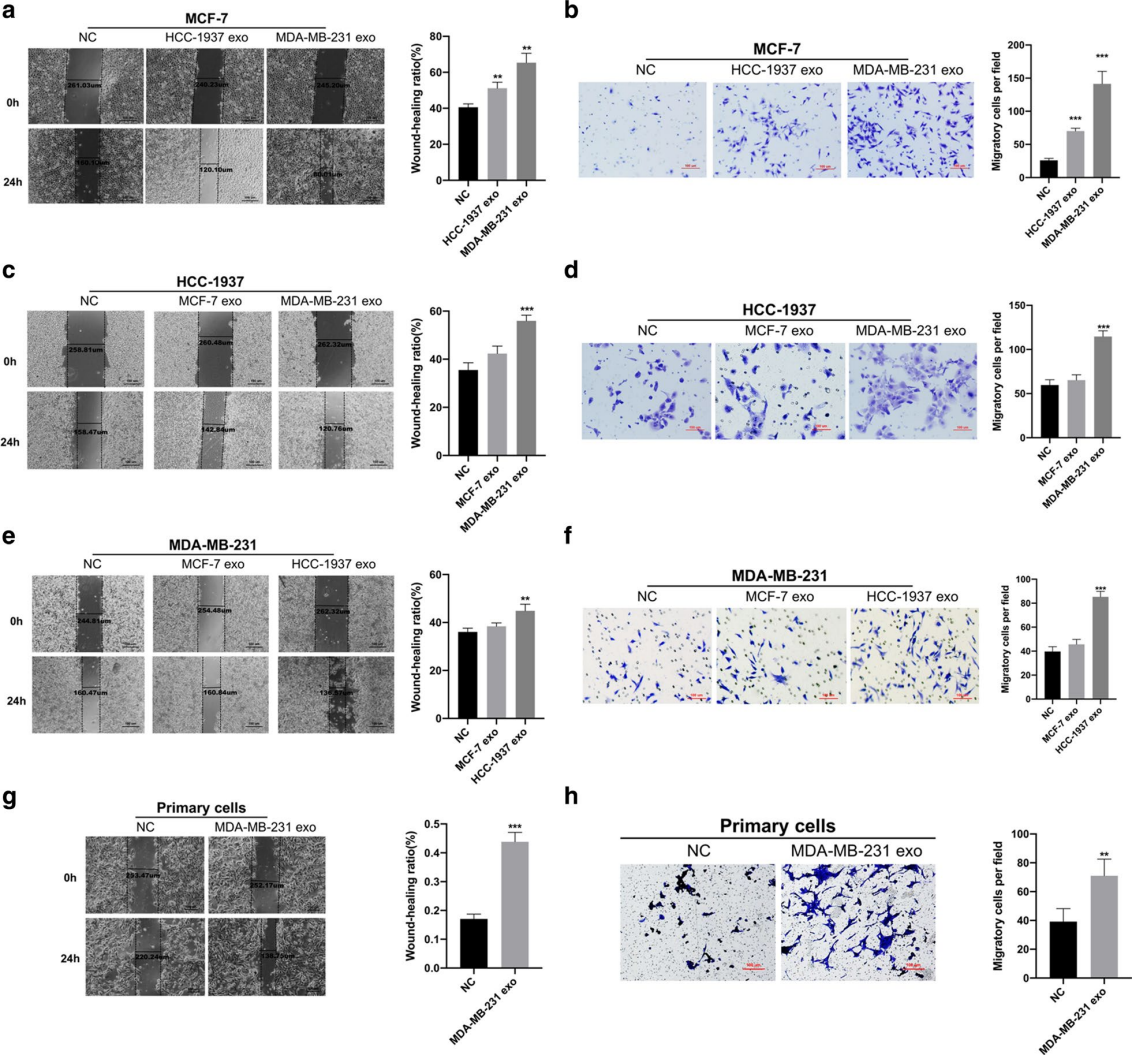
Exosomes from metastatic breast cancer cells promoted cancer cell migration and invasion.** a** Wound-healing cell migration analyses in non-metastatic MCF-7 cells treated with exosomes isolated from conditioned media of metastatic cells (HCC-1937 and MDA-MB-231). **b** Transwell cell invasion analyses of MCF-7 cells treated with exosomes isolated from HCC-1937 and MDA-MB-231 cells. **c**, **d** Comparison of the effect of exosomes from MCF-7 and MDA-MB-231 cells on the cell migration (**c**) and invasion (**d**) in HCC-1937 cells. **e**, **f** Effect of exosomes from MCF-7 and HCC-1937 on the cell migration and invasion ability of MDA-MB-231 cells examined by wound-healing assay (**e**) and Transwell invasion assay (**f**). **g**, **h** Effect of exosomes from MDA-MB-231 cells on primary breast cancer cells assessed by wound-healing cell migration (**g**) and Transwell invasion (**h**) assays. Each experiment was repeated at least three individual times, and data were presented as mean ± SD. Scale bar: 100 μm, **p < 0.01, ***p < 0.001 compared with control

HCC-1937 and MDA-MB-231 cells are metastatic cells, while MCF-7 cells are non-metastatic cells. Thus, to evaluate whether exosomes can promote migration and invasion in tumor cells, we treated breast cancer cells with exosomes from other tumor cells. We checked the cell wound-healing and Transwell invasion ability of the non-metastatic MCF-7 cells treated with exosomes isolated from the conditioned media of HCC-1937 and MDA-MB-231 cells. The analysis suggested that treatment with exosomes obtained from HCC-1937 and MDA-MB-231 cells could significantly improve the cell migration (Fig. 2a) and cell invasion ability of the MCF-7 cells (Fig. 2b) than that with control group. In contrast, exosomes from MCF-7 cells had less effect on the cell migration and invasion ability of HCC-1937 and MDA-MB-231 cells (Fig. 2c–f). Further, to investigate whether exosomes isolated from highly metastatic cells have an effect on migration and invasion ability of primary breast cancer cells, exosomes from MDA-MB-231 cells were added to the media of primary cells isolated from 5 patients with primary breast cancer. We observed that exosomes from MDA-MB-231 cells could increase the migration and invasion ability of all the primary breast cancer cells (Fig. 2g, h). Thus, these results suggested that exosomes secreted by cancer cells with different metastatic ability showed varying effectivity, and exosomes from highly metastatic cells could significantly promote cell migration and invasion in cell lines and primary tumor cells.

### MiR-7641 was enriched in metastatic exosomes and could promote tumor cell proliferation and invasion

**Fig. 3:**
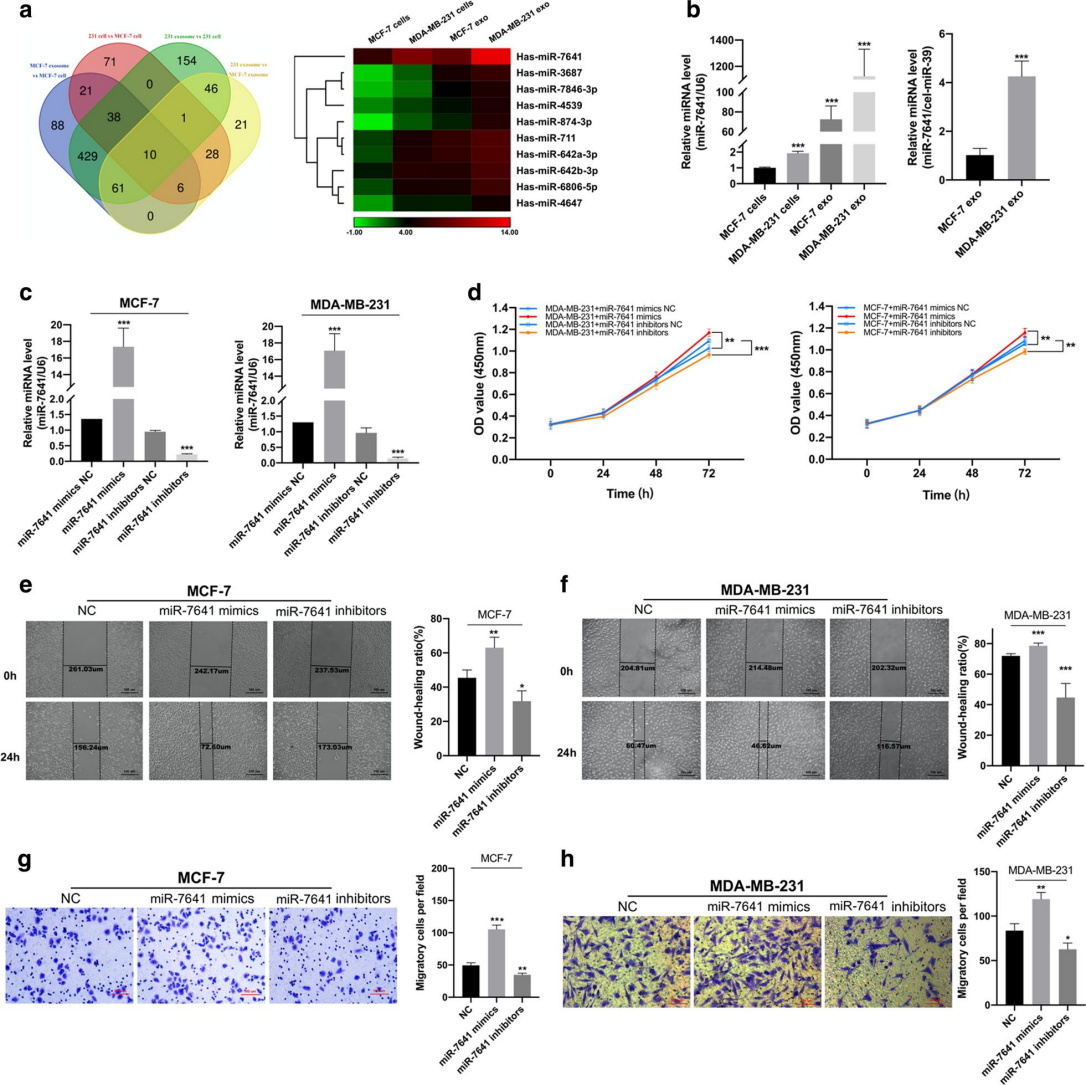
MiR-7641 was enriched in exosomes from metastatic breast cancer cells and promoted cancer cell proliferation and invasion.** a** The miRNA profiles of MCF-7 and MDA-MB-231 cells and their respective exosomes were analyzed by microarrays. Overlapping results of up-regulated miRNAs in indicated groups were analyzed and 10 miRNAs were found to be up-regulated in all the comparisons (MCF-7 exosomes *vs.* MCF-7 cells, MDA-MB-231 cells *vs.* MCF-7 cells, MDA-MB-231 exosomes *vs.* MDA-MB-231 cells, and MDA-MB-231 exosomes *vs.* MCF-7 exosomes). **b** qRT-PCR based validation of microarray analysis for miR-7641 highly enriched in metastatic cells and exosomes. The levels of miR-7641 in exosomes from MDA-MB-231 and exosomes from MCF-7 were also validated by qRT-PCR analysis using cel-miR-39 as external control. **c** qRT-PCR analysis of levels of miR-7641 in MCF-7 and MDA-MB-231 cells transfected with miR-7641 mimics and inhibitors, respectively. **d** CCK-8 assay-based cell proliferation analysis of MCF-7 and MDA-MB-231 cells transfected with miR-7641 mimics and inhibitors, respectively. **e**, **f** The wound-healing assay based cell migration analysis of MCF-7 (**e**) and MDA-MB-231 (**f**) cells transfected with miR-7641 mimics and inhibitors, respectively. **g**, **h** Transwell invasion assay analysis performed in MCF-7 (**g**) and MDA-MB-231 cells (**h**) transfected with miR-7641 mimics and inhibitors, respectively. Each experiment was repeated at least three independent times, and results were presented as mean ± SD. 231: MDA-MB-231, exo: exosomes, scale bar: 100 μm, *p < 0.01, **p < 0.01, ***p < 0.001 compared with control

Next, we investigated the essential components in the tumor-derived exosomes that conferred the tumor-promoting effect. We selected the metastatic MDA-MB-231 and non-metastatic MCF-7 for identifying the key miRNAs. The miRNA profiles of endogenous miRNAs and those in exosomes derived from these two cell lines were obtained using microarray analysis. Only miRNAs that were differentially expressed with at least twofold change were considered as significant candidates. Further, we used the following criteria for screening essential miRNAs that might epigenetically modify the features of target cells: (1) increased levels in exosomes derived from metastatic breast cancer cells (MDA-MB-231) than in those derived from non-metastatic breast cancer cells (MCF-7); (2) enrichment in exosomes than in their cells of origin; (3) increased levels in MDA-MB-231 cells than that in MFC-7 cells. As shown in Fig. 3a, 10 miRNAs (miR-7641, miR-3687, miR-7846-3p, miR-4539, miR-874-3p, miR-711, miR-642a-3p, miR-642b-3p, miR-6806-5p, and miR-4647) qualified these criteria, and were further subjected to validation by qRT-PCR analysis. Of these 10 miRNAs, miR-7641 was found to be significantly differentially expressed at both endogenous and secretory levels (Fig. 3b). Moreover, the increased levels of miR-7641 in exosomes from MDA-MB-231 than those from MCF-7 were confirmed with qRT-PCR analysis using both internal control *U6* and external control cel-miR-39 (Fig. 3b).

Next, to investigate the role of miR-7641, we transfected MCF-7 and MDA-MB-231 cells with miR-7641 mimics and inhibitors. Treatment with miR-7641 mimics could significantly increase the levels of miR-7641 in MCF-7 and MDA-MB-231 cells, while miR-7641 inhibitors could decrease the expression of miR-7641 in these cells (Fig. 3c). Further, the CCK-8 assay showed that miR-7641 mimics could significantly increase the cell proliferation rate in MCF-7 and MDA-MB-231 cells, and miR-7641 inhibitors could significantly decrease cell proliferation in these cells than that with negative control (Fig. 3d). Additionally, the wound-healing analysis suggested that miR-7641 mimics increased tumor cell migration; and, miR-7641 inhibitors decreased migratory ability of MCF-7 and MDA-MB-231 cells (Fig. 3e). Furthermore, similar results were obtained with the Transwell invasion assays (Fig. 3f).

Taken together, these results demonstrated that miR-7641, identified in microarray analysis and validated by qRT-PCR analysis, played an important role in breast cancer cell proliferation, migration, and invasion.

### MiR-7641 could be transferred via exosomes to promote recipient tumor cell proliferation and invasion

**Fig. 4:**
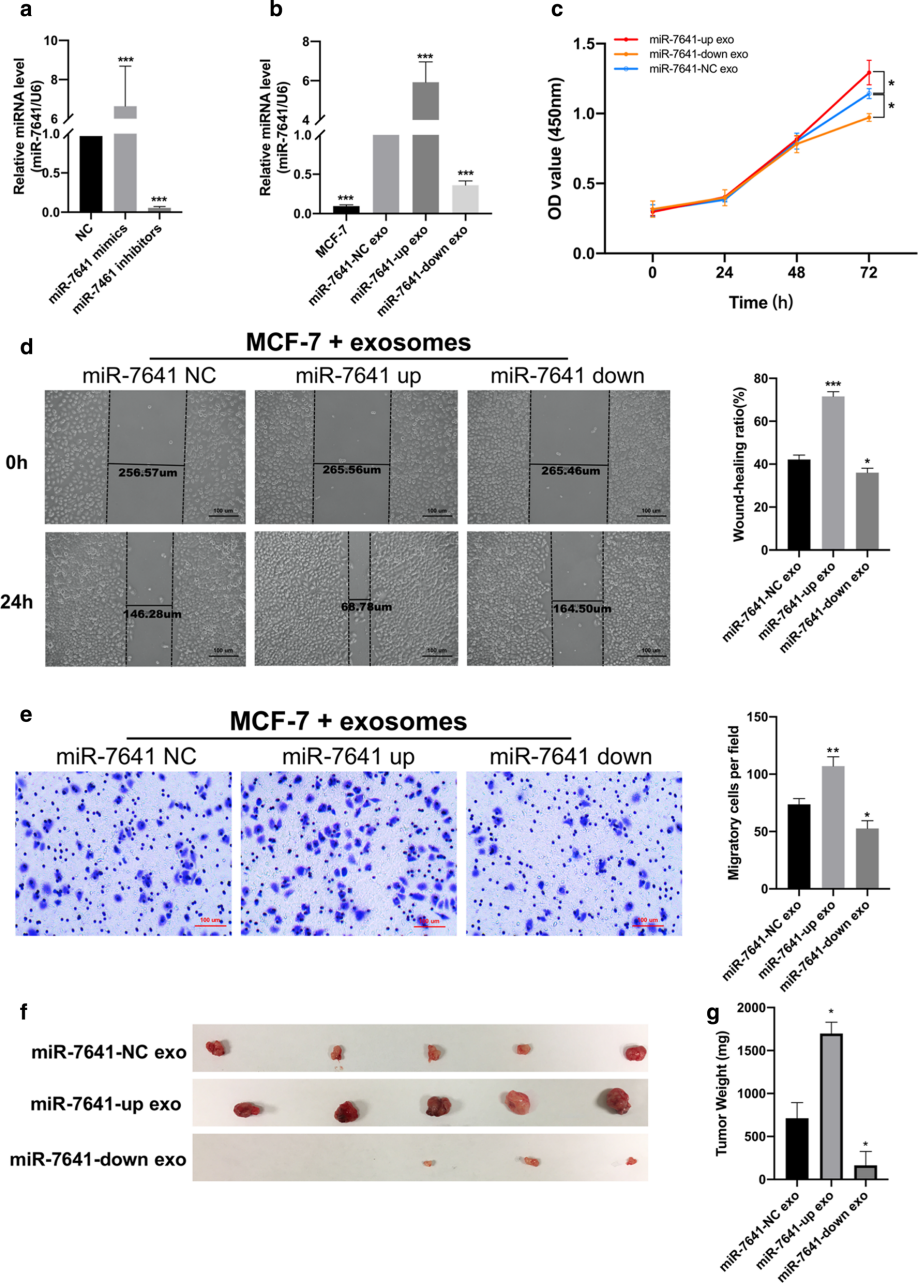
Cancer-derived exosomal miR-7641 promoted breast tumor cell growth and progression.** a** qRT-PCR analysis of miR-7641 in exosomes isolated from MDA-MB-231 cells transfected with miR-7641 mimics or inhibitors. **b** qRT-PCR analysis of endogenous miR-7641 levels in MCF-7 cells incubated with exosomes with varying miR-7641 levels. **c** CCK-8 based cell proliferation analysis in MCF-7 cells treated with exosomes with varying miR-7641 levels. **d** Wound-healing assay-based analysis of cell migration in MCF-7 cells treated with exosomes with varying miR-7641 levels. **e** Transwell invasion assay analysis in MCF-7 cells treated with exosomes with varying levels of miR-7641. **f** Tumorigenicity analysis in xenograft mice (*N* = 15; 5 mice in each group) injected with exosomes with varying levels of miR-7641 levels. Each experiment was repeated at least three individual times and data were presented as mean ± SD. exo: exosomes, scale bar: 100 μm, *p < 0.01, **p < 0.01, ***p < 0.001 compared with control

To validate whether presence of miR-7641 in exosomes is essential for their effect on tumor cells, we transfected MDA-MB-231 cells with miR-7641 mimics and inhibitors to produce exosomes with up-regulated and down-regulated levels of miR-7641, respectively. As shown in Fig. 4a, exosomes isolated from media of MDA-MB-231 cells transfected with miR-7641 mimics (miR-7641-up exosomes) had significantly increased levels of miR-7641; whereas exosomes from media of cells transfected with miR-7641 inhibitors (miR-7641-down exosomes) had significantly low levels of miR-7641 than that in exosomes from media of cells transfected with the negative control (miR-7641-NC exosomes). Further, we incubated MCF-7 cells for 24 h with these three kinds of exosomes and checked the endogenous levels of miR-7641 by qRT-PCR. The analysis suggested that MCF-7 cells treated with miR-7641-up exosomes showed increased levels of miR-7641, while those treated with miR-7641-down exosomes showed decreased levels of miR-7641 than that in cells treated with miR-7641-NC exosomes (Fig. 4b). Additionally, to investigate whether miR-7641 transferred via exosomes remain functional in the recipient cells, MCF-7 cells treated with exosomes containing varying levels of miR-7641 were subjected to proliferation, wound-healing, and Transwell invasion assays. MCF-7 cells treated with miR-7641-up exosomes showed significant increase in levels of cell proliferation, migration, and invasion than that in cells treated with miR-7641-NC exosomes (Fig. 4c–e). However, treatment of MCF-7 cells with miR-7641-down exosomes showed significantly low levels of cell proliferation, migration, and invasion than that in cells treated with miR-7641-NC exosomes.

Next, to confirm whether cancer-derived exosomal miR-7641 also plays a role in cancer progression in vivo, we injected the three kinds of exosomes into xenograft mice and analyzed the xenograft tumor volume. The xenograft tumors treated with miR-7641-up exosomes showed the highest tumorigenicity, while those treated with miR-7641-down exosomes showed the lowest tumorigenicity (Fig. 4f).

Thus, these results suggest that cancer-derived exosomal miR-7641 can be transferred to breast tumor recipient cells, and promote proliferation and invasion in those cells, in vitro and in vivo.

### Exosomal miR-7641 in plasma correlated with breast cancer metastasis

**Fig. 5:**
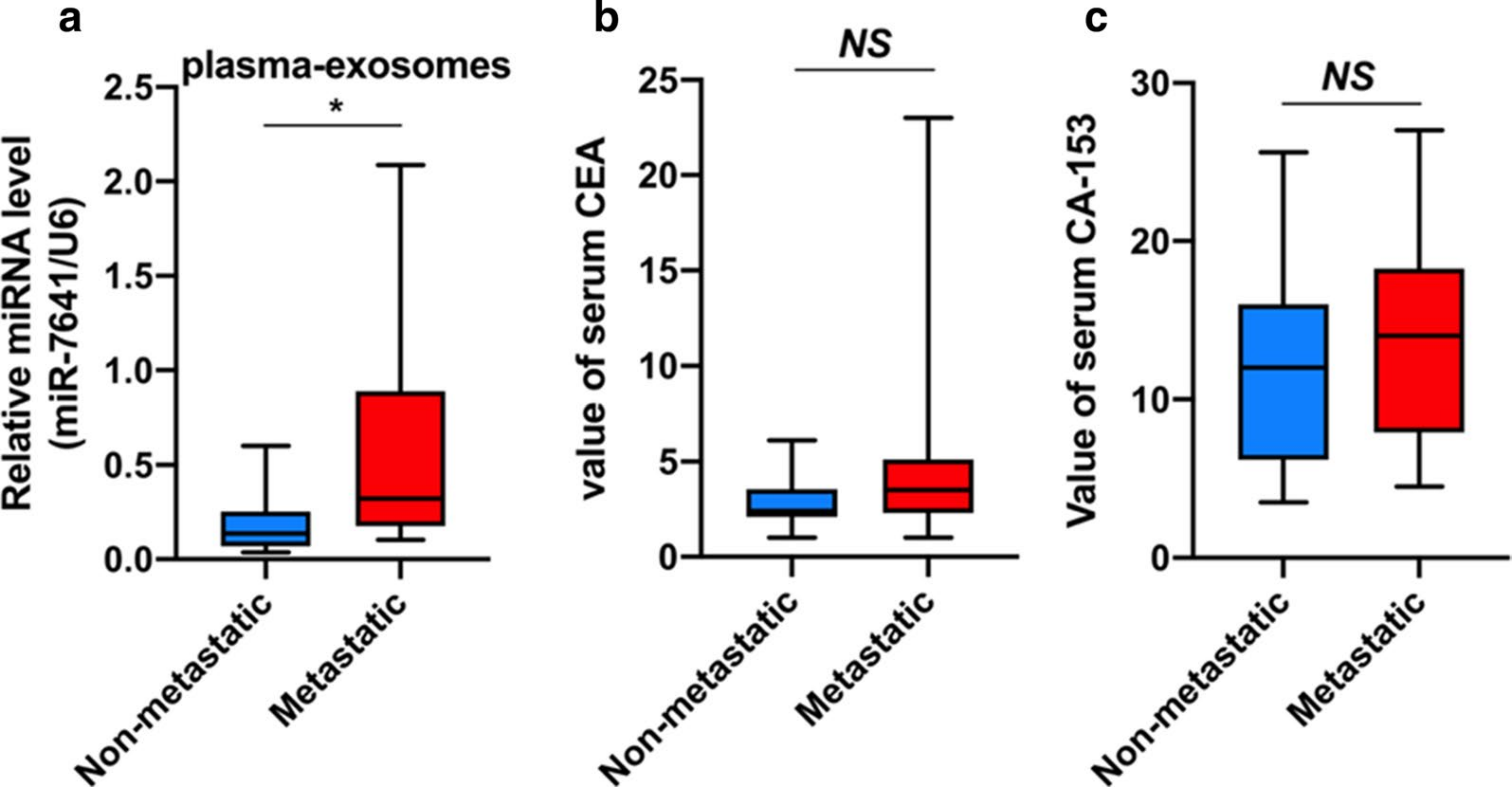
Exosomal miR-7641 in plasma was associated with metastasis in breast cancer patients.** a** qRT-PCR analysis of miR-7641 in plasma samples collected from breast cancer patients with distant metastasis (*N* = 13) and those without metastasis (*N* = 15). **b**, **c** Analysis of serum levels of CEA (**b**) and CA 153 (**c**) in breast cancer patients with or without distant metastasis. Data were presented as mean ± SD. CEA: carcinoembryonic antigen, CA 153: carbohydrate antigen 153, NS: not significant, *p < 0.01

Further, to assess the correlation between exosomal miR-7641 and metastasis, we collected exosomes isolated from breast cancer patients with or without distant metastasis; and, analyzed the levels of miR-7641 in these exosomes by qRT-PCR. As shown in Fig. 5a, breast cancer patients with distant metastasis showed significantly elevated levels of miR-7641 than in patients without metastasis. However, there was no significant difference in the serum levels of the two commonly used tumor biomarkers—carcinoembryonic antigen (CEA) and carbohydrate antigen (CA) 153—between breast cancer patients with or without distant metastasis (Fig. 5b, c).

Taken together, these results showed that miR-7641 could promote tumor cell proliferation and invasion, and miR-7641 could be transferred via exosomes to play a pro-invasion role in recipient tumor cells. Additionally, our findings also indicated that exosomal miR-7641 was a better plasma biomarker for predicting tumor metastasis than the traditional CEA and CA153 serum markers.

### MiR-7641 correlates with breast cancer survival and targets several biological processes

**Fig. 6:**
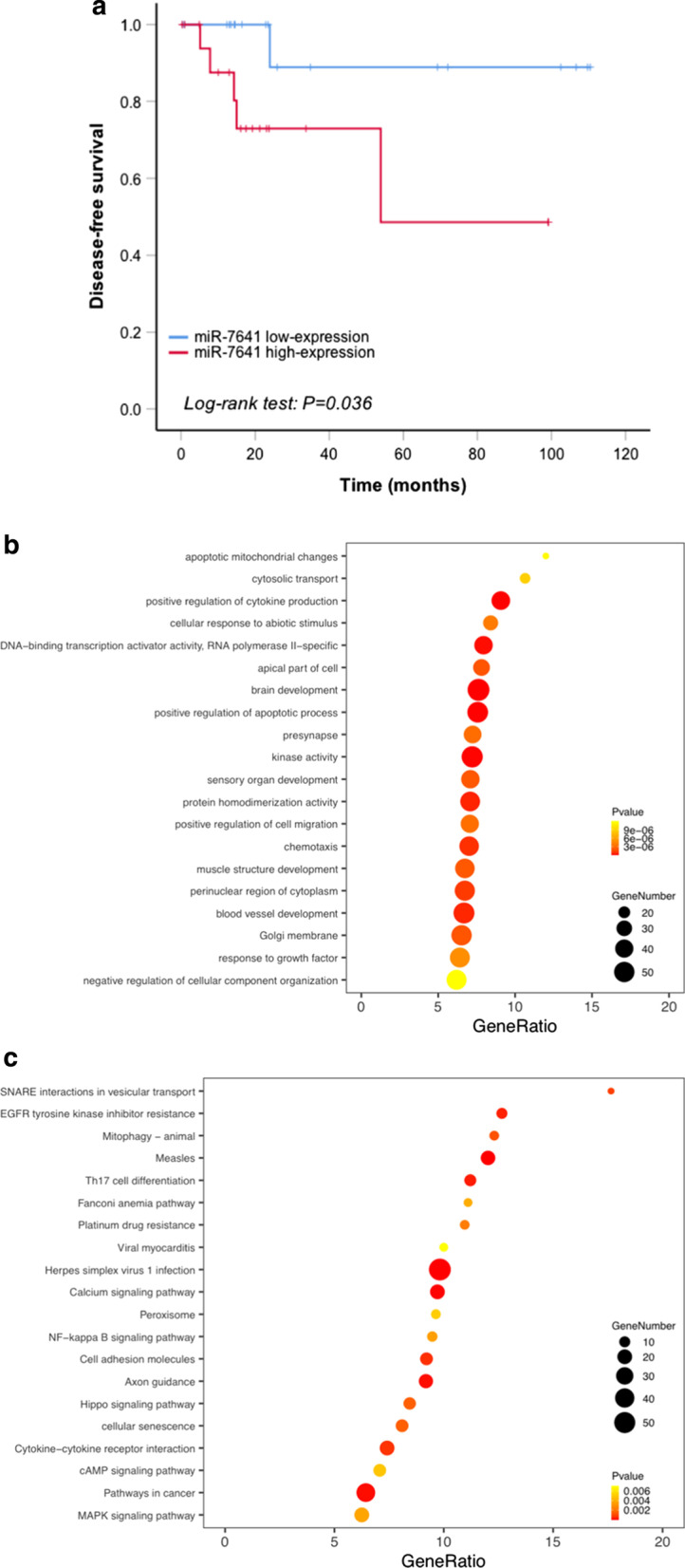
MiR-7641 correlates with breast cancer survival and targets several biological processes.** a** Data from TCGA database showed high level of expression of miR-7641 is correlated with unfavorable DFS in patients with breast cancer (*p* = 0.036). **b** Bubble plot of GO enrichment analysis of target genes of miR-7641. **c** Bubble plot of KEGG enrichment analysis of target genes of miR-7641

Data from TCGA database showed the expression level of miR-7641 was correlated with DFS of patients with breast cancer (Fig. 6a), with the high-expression group showing a more unfavorable outcome of survival (p = 0.036). An overlapped collection of 2528 target genes were predicted by the three algorisms of TargetScan, miRWalk, and miRanda databases. GO and KEGG enrichment analysis suggested that miR-7641 was closely related with the biological processes including apoptotic mitochondrial changes, cytosolic transport and cytokine production according to GO enrichment analysis (Fig. 6b), and SNARE interaction in vesicular transport, GFR tyrosine kinase inhibitor resistance, and mitophagy by KEGG enrichment analysis (Fig. 6c).

## Discussion

Several studies have described exosomes as important mediators of intercellular communication in breast cancer progression and metastasis. In this study, we confirmed the characteristics and functional heterogeneity in exosomes derived from breast cancer cells with varying metastatic potential. We identified that miR-7641 could promote breast cancer cell proliferation and invasion, and that its ability to cause epigenetic modulation could affect recipient cells after transportation via exosomes. Moreover, exosomal miR-7641 in the plasma was also found to be elevated in breast cancer patients with distant metastasis. To the best of our knowledge, this is the first report to elucidate the functions of exosomal miR-7641 in breast cancer.

The results support the recent concept that exosomes can act as carriers of functional messages[15]. Additionally, miRNAs are efficient post-transcriptional regulators and are selectively enriched in exosomes[10–14]. A few miRNAs have already been shown to promote tumor progression and metastasis via transfer of exosomes in a variety of cancer types[19]. We hypothesized that the functional heterogeneity of exosomes originated from differences in the abundance of their cargoes, and that miRNAs might be the most important components of “messages”. In the present study, miR-7641 was identified to be the most significant differentially expressed miRNA at both endogenous and secretory levels in highly metastatic breast cancer cells. Furthermore, the results showed miR-7641 to be enriched in exosomes compared to their cells of origin. This special enrichment of miR-7641 was previously reported in colorectal cancer, where miR-7641 was found to be highly expressed in metastatic colorectal cancer cells and enriched in exosomes secreted from cells, but was undetectable in non-metastatic isogenic cancer cells[20].

MiR-7641 is a hitherto poorly investigated miRNA. It was first reported that miR-7641 could modulate the expression of CXCL1 during endothelial differentiation derived from human embryonic stem cells[21]. The tumor-promoting function of miR-7641 observed in our study is consistent with previous results in gastric cancer[22] and bladder cancer[23]. It was also reported that inhibition of miR-7641 reduced cell proliferation in breast and colon cancer cell lines, and enhanced efficiency of doxorubicin[24]. Moreover, our results showed that the tumor-promoting capacity of miR-7641 could be transferred to recipient cancer cells via exosomes, and miR-7641 also increased tumor growth in vivo.

The results indicated that breast cancer patients with distant metastasis had significantly increased levels of miR-7641 in plasma than that in patients without metastasis. Also, bioinformatics analysis has suggested that miR-7641 is correlated with breast cancer survival, and several important cellular and biological processes are closely targeted by miR-7641. Gallo et al.[25] found that majority of the miRNAs detectable in serum were concentrated in exosomes. Moreover, miRNAs are more stable than mRNAs, and thus less prone to external contamination during sample processing, especially in body fluid tests, which showed great prognostic potential in clinical practice. Pan et al.[26] revealed that miR-7641, as a cerebrospinal fluid miRNA, has potential value for diagnosing and monitoring leptomeningeal metastasis. These characteristics make them excellent candidates as circulating biomarkers. Thus, exosomal miRNAs are gaining recognition as diagnostic and prognostic biomarkers in liquid biopsies. Several efforts have been made in this field and a few exosomal miRNAs in plasma have been successfully identified as useful diagnostic biomarkers in breast cancer and other cancers[27–29]. Therefore, exosomal miR-7641 can be indicated as a promising non-invasive diagnostic biomarker.

## Conclusion

In conclusion, this study demonstrated that metastatic capability of exosomes was related to their parental cells. Additionally, miR-7641 was identified as an important component of exosomes that could promote breast tumor progression and metastasis via intercellular communication. Moreover, cancer-derived exosomal miR-7641 was positively correlated with breast cancer metastasis. This study added new insights to the molecular mechanisms underlying cell–cell communication during breast tumor progression and metastasis, contributing to improved diagnostic and therapeutic strategies in breast cancer. However, the mechanisms by which cancer-derived miR-7641 regulates downstream signaling pathways and its role in communicating with stromal cells in the tumor microenvironment remain to be further investigated.

## Supplementary Information

**Additional file 1: Table S1**. The primers used for qRT-PCR analysis were purchased from RiboBio (Guangzhou, China) and the catalog numbers of miRNAs were listed as following.

## Data Availability

The datasets supporting the conclusions of this article are included within the article and the additional file. More supporting data is available under reasonable request.

## Abbreviations

miRNA: MicroRNA
PUMCH: Peking Union Medical College Hospital
DMEM: Dulbecco’s modified Eagle’s Medium
FBS: Fetal bovine serum
PBS: Phosphate-buffered saline
TEM: Transmission electron microscopy
NC: Negative control
CEA: Carcinoembryonic antigen
CA153: Carbohydrate antigen
