# Supplementary material for: Cancer-derived exosomal miR-7641 promotes breast cancer progression and metastasis
Source: Cell Commun Signal. 2021 Feb 22;19:20. doi: 10.1186/s12964-020-00700-z (PMC7898766; doi:10.1186/s12964-020-00700-z)
Supplement: Supplementary file 2 — Additional file 1: Table S1. The primers used for qRT-PCR analysis were purchased from RiboBio (Guangzhou, China) and the catalog numbers of miRNAs were listed as following. [file 12964_2020_700_MOESM2_ESM.docx]

Supplementary Table S1

The primers used for qRT-PCR analysis were purchased from RiboBio (Guangzhou, China) and the catalog numbers of miRNAs were listed as following.

| microRNA | Stem-loop RT primer | forward primer |
| --- | --- | --- |
| U6 | SSD0904071008 | SSD0904071006 |
| cel-miR-39 | SSD1083145001 | SSD1083145002 |
| hsa-miR-7641 | mir8001909 | mir8001910 |
| hsa-miR-3687 | SSD13422385420 | SSD13422385421 |
| hsa-miR-7846-3p | miR8004598 | miR8004599 |
| hsa-miR-4539 | SSD1417843060 | SSD1417843061 |
| hsa-miR-874-3p | SSD809230649 | SSD090525062 |
| hsa-mir-711 | miR8004596 | miR8004597 |
| hsa-miR-642a-3p | SSD1352110497 | SSD1352110498 |
| hsa-miR-642b-3p | MIR8002333 | MIR8002334 |
| hsa-miR-6806-5p | MIR8004782 | MIR8004783 |
| hsa-miR-4647 | SSD1353927060 | SSD1353927061 |
